# Dietary Patterns and the Prevalence of Noncommunicable Diseases in the PURE Poland Study Participants

**DOI:** 10.3390/nu15163524

**Published:** 2023-08-10

**Authors:** Dorota Różańska, Krzysztof Kujawa, Andrzej Szuba, Katarzyna Zatońska, Bożena Regulska-Ilow

**Affiliations:** 1 Department of Dietetics and Bromatology, Wroclaw Medical University, 50-556 Wroclaw, Poland; 2 Statistical Analysis Center, Wroclaw Medical University, 50-368 Wroclaw, Poland; 3 Department of Angiology, Hypertension and Diabetology, Wroclaw Medical University, 50-556 Wroclaw, Poland; 4 Division of Population Studies and Prevention of Non-Communicable Diseases, Department of Population Health, Wroclaw Medical University, 50-372 Wroclaw, Poland

**Keywords:** noncommunicable diseases, cardiovascular disease, hypertension, diabetes, impaired fasting glucose, dietary patterns

## Abstract

The aim of the study was to analyze the association between dietary patterns (DP) identified in the group of Lower Silesia (Poland) inhabitants and the prevalence of selected noncommunicable diseases, such as total cardiovascular diseases (CVD), hypertension, diabetes, impaired fasting glucose (IFG), visceral obesity, and excessive body weight. This study involved 2023 subjects aged 35–70 years, from Wroclaw and surrounding villages. The assessment of food intake in the study group was assessed using the standardized Food Frequency Questionnaire. Dietary patterns were identified using the principal components analysis (PCA) with varimax rotation. Three dietary patterns were identified in the study group: unhealthy, healthy, and traditional. The prevalence of hypertension, diabetes, IFG, and visceral obesity decreased across the quartiles of healthy DP. Prevalence of IFG increased across the quartiles of unhealthy DP, but the prevalence of hypertension decreased. When the diet was more adherent to the traditional DP the prevalence of CVD, diabetes, IFG, visceral obesity, obesity, and being overweight was higher. DP abundant in fruits, vegetables, seeds, nuts, raisins, and unrefined grains, named “healthy dietary pattern”, had a beneficial association with lower prevalence of selected noncommunicable diseases. DP abundant in meat products, but poor in fruits and vegetables were positively associated with higher prevalence of total CVD, diabetes, IFG, excessive body weight, and visceral obesity.

## 1. Introduction

In the middle of the twentieth century, infectious diseases as the main cause of morbidity and death gave place to the diseases with metabolic disorders, mainly atherosclerosis and its chronic and acute complications. A lot of attention was paid to the lifestyle risk factors, the components of which are diet and physical activity. Research on the relationship between nutrition and the risk of chronic diseases was initiated by the Framingham Heart Study, which led to the identification of risk factors for cardiovascular diseases [1]. In the INTERHEART study, ten risk factors responsible for 90% of cases of acute complication of atherosclerosis, i.e., myocardial infarction, were specified [2]. Two editions of the Seven Countries Study provided the first evidence of an association between nutrition and coronary heart disease and gave the basis for recommending a Mediterranean diet for the prevention of cardiovascular diseases [3]. Another milestone on the way to a better understanding of the importance of nutrition in heart prevention was the “Lyon Diet Heart Study” [4]. The Mediterranean diet, with lower carbohydrate and higher fat content, is the only dietary approach that has been shown to reduce the risk of cardiovascular events (such as myocardial infarction or stroke) or cardiovascular death in both primary and secondary prevention, what was proved in the PREDIMED [5] and the Lyon Diet Heart Study [4], respectively.

Systematic analysis prepared based on the data from 195 countries across the Global Burden of Disease (GBD) Study 2017 gives evidence that there is a strong association between diet and health. It was found that globally, in 2017, dietary risks factors were responsible for 11 million deaths (22% of all deaths among adults) and 255 million disability-adjusted life-year (15% of all DALYs among adults). The leading cause of diet-related deaths, amounting to 10 million, was cardiovascular disease (CVD). It was also responsible for 207 million DALYs. Second place among diet-related deaths was assigned to cancers, while the third place to type 2 diabetes. It is also worth noting that 45% of total diet-related deaths and 70% of total diet-related DALYs was observed for adults below 70 years old. The leading dietary risk factors were high intake of sodium followed by low intake of whole grains and low intake of fruits. It was found that globally the intake of almost all healthy foods and nutrients did not reach the optimal level but, on the other hand, the intake of all unhealthy foods and nutrients was higher than the optimal level. Among healthy foods the highest disproportion between the observed and optimal intake was noticed for nuts and seeds, milk, and whole grains. The highest exceedance of the recommendations among unhealthy foods was observed for sugar-sweetened beverages, processed meat, sodium, and red meat [6]. Based on the data published by the Statistics Poland in the report “Health status of population in Poland in 2019” noncommunicable diseases are also often diagnosed in Poland. The prevalence of high blood pressure, diabetes, coronary artery disease as well as stroke and myocardial infraction increase with age. In 2019, in Poland, the prevalence of high blood pressure was 14.9% among 40–49 years old people, 35.5% among 50–59 years old people, and 66.5% among those aged 70–79 years old. The prevalence of diabetes in these age groups was 2.7%, 8.5%, and 26.5%, respectively. Among people aged 40–49 and 50–59 years old the prevalence of coronary artery disease was 1.3% and 5.1% but among people aged 70–79 years old it amounted to 26.6% and the prevalence of stroke and myocardial infraction in this group was 6.1% and 8.2%, respectively. Excessive body weight was found in about 60% of people aged 40–49 years old and in about 70% of people aged 70–79 years old [7]. According to the data published by the National Institute of Public Health—National Institute of Hygiene in the report “Health status of Polish population and its determinants 2020” based on the analyses for 2019, performed as part of the GBD2019 study, a level of significance of behavioral risk factors, accounting in Poland for 52% of deaths from cardiovascular diseases, 42% deaths from cancer, and 44% of all-cause mortality, which is higher than the global level (49%, 37% and 38%, respectively). Risk factors posing the most serious threat to life in Poland include hypertension, accounting for 22.3% of deaths, tobacco (20.4%, active smoking—19.1%), and unhealthy diet (20.0%). In 2010–2018, the Polish population consumed too few vegetables and fruit, and too much red meat and processed meat, as compared to the recommendations. The excessive proportion of energy from fats, including saturated fatty acids, and too little energy from carbohydrates were also observed in their diets. In the diets of the Polish adult population the deficiencies of minerals and vitamins, in particular, calcium, magnesium, potassium, vitamin C and D, and folates were observed. On the other hand, there was too high a content of sodium in the diet [8]. It is worth noting that taking into account the socio-demographic index (SDI), developed by GBD researchers, which incorporates the results of the analysis of the total fertility rate under 25, secondary education for those aged 15 and over and per capita income distributed over time, Poland belongs to countries with a medium-high SDI (SDI = 0.80), with a minimum value of 0.77 in the Warmińsko-Mazurskie voivodship and a maximum value of 0.84 in the Mazowieckie voivodship [9]. Based on the GBD analysis it was shown that high-middle and middle SDI countries were at the greatest risk of deaths and DALYs from high intake of sodium, whereas high and low-middle SDI countries had the greatest risk caused by a diet low in whole grains. It was also shown that high sodium, low whole grains, low fruit, and low nuts and seeds intake were the same four leading dietary risks in countries at all levels of SDI other than low SDI [6].

However, it is well known that human nutritional status depends on the synergistic effect of the combination of all consumed food, and assessment of the relations between diet quality and health outcomes are nowadays mainly based on various diet quality scales. Often used are those that characterize dietary patterns using a priori scoring systems of indices, such as the Healthy Eating Index 2015 [10], Healthy Diet Indicator [11], or the Dietary Approaches to Stop Hypertension (DASH) diet score [12]. Many authors also use a different modification of Mediterranean diet scales, which are for example based on the median intake of food groups [11,13] or on the precisely given cut-off points [14]. Worth noting is evidence that diet, in relation to health status, should be treated as a total effect of an intake of different food as has been published by Trichopoulou et al. [15]. Based on the population-based, prospective study involving 22,043 adults, where the median of follow-up was 44 months, it was shown that higher adherence to the Mediterranean diet was associated with a reduction in total mortality, as well as in deaths due to coronary heart disease and cancer. However, no significant associations were observed between individual food groups contributing to the Mediterranean-diet score and total mortality, except for the fruits + nuts and monounsaturated to saturated fatty acids ratio [15]. There are also methods used to assess dietary patterns which are calculated a posteriori, for example principal component analysis (PCA), which is also often used in epidemiological studies [16,17].

The aim of the herein study was to analyze the association between dietary patterns identified in the group of Lower Silesia inhabitants and the prevalence of selected noncommunicable diseases, such as: total cardiovascular diseases, hypertension, diabetes, impaired fasting glucose, visceral obesity, and excessive body weight.

## 2. Materials and Methods

### 2.1. Study Group

The study group included participants of the Polish arm of the Prospective Urban Rural Epidemiology (PURE) study, which is global, epidemiological, and cohort. The PURE study is conducted in a group of over 150,000 adults from 17 low-, middle-, and high-income countries. Detailed objectives and assumptions of the PURE study are described elsewhere [18,19].

This study involved 2023 subjects (1274 women and 749 men) aged 35–70 years, from Wroclaw (*n* = 1200) and surrounding villages in Lower Silesia (*n* = 823). Participants were recruited through press and radio advertisements in the years 2007–2009. Written informed consent was obtained from the participants for all study procedures. Participation in the study was voluntary.

### 2.2. Measurements (Outcomes)

In this study we have analyzed the association between dietary patterns and the prevalence of the following noncommunicable diseases: total cardiovascular diseases (CVD), hypertension, diabetes, impaired fasting glucose (IFG), visceral obesity, and excessive body weight (overweight + obesity). The data about CVD incidents were based on the interviews collected with participants about their medical history. Hypertension prevalence was assessed based both on the blood pressure measurement (≥140 and/or ≥90 mm Hg) and declaration about hypertension occurrence in the interview. Diabetes diagnosis was made based on a fasting glucose level ≥ 126 mg/dl or declaration about diabetes occurrence in the interview. IFG was diagnosed when the fasting glucose level was between 100–125 mg/dl with no statement about diabetes in the interview. Waist circumference ≥80 cm for women and ≥94 cm for men was a criterion for visceral obesity diagnosis. For the group with excessive body weight, participants with a body mass index (BMI) ≥ 25 kg/m^2^ were classified.

### 2.3. Dietary Assessment

The assessment of food intake in the study group was assessed using the standardized Food Frequency Questionnaire (FFQ), which was developed and validated for this population within the PURE project [20]. FFQ includes questions about 154 food items: milk and dairy products (20 products), fruits (13 products), vegetables (33 products), meat and eggs (23 products), breads and cereals (9 products), mixed foods (21 products), drinks (17 products), snacks (18 products), and 7 types of oils. Each respondent has to answer the question: how often during the year preceding the study he/she consumed particular products. The frequency of consumption was given in relation to the average serving size, expressed in household measures such as 1 glass, 1 teaspoon, 1 plate, 1 slice or piece, and 1 medium fruit. The study participants could choose one of the following frequencies of consumption during a day (once, 2–3 times, 4–5 times, 6 or more times a day), during a week (once, 2–4 times, 5–6 times a week), or a month (never or less than once a month, 1–3 times a month). Frequency of consumption of each food item expressed in household measures was computed into daily intake (g/day).

### 2.4. Identification of Dietary Patterns

Dietary patterns (DP) were identified using the principal components analysis (PCA) with varimax rotation. Analysis was based on the frequency of daily intake of 22 food groups. Food items from the FFQ were categorized into 22 groups based on their similar composition and nutritional value as follows: milk and low-fat dairy products, high-fat cheese and cream, margarines and mayonnaise, animal fats, eggs, fish, unrefined grains, refined grains, “mixed dishes”, soups, alcohol, beverages, sweets, sugar and honey, nuts and seeds and raisins, fruits, juices, vegetables, tea and coffee, red and processed meat, poultry, potatoes and chips. The food group called “mixed dishes” includes dishes composed of products belonging to different groups, for example vegetables and meat or grains and meat or dairy. The “mixed dishes” included in the FFQ were typical for Polish cuisine, such as beans bakes with meat in tomato sauce, cabbage leaves stuffed with meat and rice in tomato sauce, dumplings with meat, sauerkraut with sausage and meat (stewed), dumplings with potato and cottage cheese, and cooked vegetable salad with mayonnaise. The average daily intake of each food group is presented in the Supplementary Materials Table S1. In the PCA factor loadings ≥ 0.5 were used as cut-off points. Names for dietary patterns were given based on the main food groups present in each of them. Quartiles of each dietary pattern determined by factor scores represents the level of adherence to DP, where the lower quartile was tantamount with low adherence and the upper quartile was tantamount with high adherence to each DP.

### 2.5. Statistical Analysis

Principal components analysis (PCA) was used to identify dietary patterns, where factor loadings ≥ 0.5 were used as cut-off points. The prevalence of selected noncommunicable diseases (categorical variables) in quartiles of DP were expressed as a number of incidents and percentage of incidents. To assess trends for the prevalence of selected noncommunicable diseases across quartiles of each DP, the Cochran–Armitage test for trend was used. Logistic regression analysis was performed with the use of the Akaike information criterion (AIC) for best predictor subset selection with the given dietary pattern (Supplementary Materials Tables S2–S7). Because we tested the impact of DP, we applied the condition that DP should be present in each model (DP have been excluded from the predictor selection). Next, the best models whose AIC differed from each other by less than 2, were averaged using Akaike weights. The consecutive quartiles, which grade the given dietary patterns (healthy, unhealthy, and traditional) were considered ordinal predictors. For such the predictors, their consecutive powers, i.e., linear (L), quadratic (Q), and cubic (C) are present in a given model, which enables us to interpret the effect of this ordinal predictor as linear or curvilinear. The variance inflation factor (VIF) was used to check lacking collinearity of the predictors with the assumption for VIF < 5 as the values indicating no predictor collinearity. The assumption of the linear relationship between explanatory variables and the logit of the response variable was checked using the Box-Tidwell test. Statistical analyses were conducted using the Statistica software (TIBCO Software Inc. 2017; version 13.) and the MuMin package [21] in the R 4.1.2 environment [22]. The level of statistical significance was set at *p* < 0.05.

## 3. Results

Based on the PCA analysis with varimax rotation, three dietary patterns were identified in the study group: unhealthy DP, healthy DP, and traditional DP (Table 1). Total percent of explained variance accounted for 33%. For the unhealthy DP, the intake of refined grains, high fat cheese and cream, margarines and mayonnaise, sweets, sugar and honey was characteristic. The consumption of fruits, vegetables, nuts, seeds, raisins, and unrefined grains was associated with the healthy DP. The dietary pattern with the highest factor loading for “mixed dishes”, which were typical for Polish cuisine, was called traditional DP. Other food groups with a factor loading ≥0.5 (cut-off point) in this DP were soups (which are also typical for Polish cuisine), fish, red and processed meat, and poultry.

Table 2, Table 3 and Table 4 show the prevalence of selected noncommunicable diseases between quartiles of healthy, unhealthy, and traditional DP, respectively. It was found that the prevalence of hypertension (*p* = 0.0003), diabetes (*p* = 0.0009), IFG (*p* = 0.0089), and visceral obesity (*p* = 0.0348) decreased across the quartiles of healthy DP (Table 2). The prevalence of IFG increased across the quartiles of unhealthy DP (*p* = 0.0037), but the prevalence of hypertension decreased (*p* = 0.0012), as is shown in Table 3. When the diet was more adherent to the traditional DP the prevalence of CVD (*p* = 0.0391), diabetes (*p* = 0.0014), IFG (*p* = 0.0000), visceral obesity (*p* = 0.0003), obesity, and being overweight (*p* = 0.0000) was higher (Table 4).

Logistic regression results performed to assess the relationship between dietary patterns and the prevalence of selected noncommunicable diseases are shown in Table 5 and Table 6. No significant association was found for CVD prevalence and dietary patterns. In a logistic regression model including age, BMI, energy intake, fiber, gender, place of residence, saturated fatty acids, and smoking, the unhealthy DP was associated with a lower risk of hypertension prevalence (OR 0.77; 95% CI 0.62–0.95; *p* < 0.05). In a model including age, energy intake, fiber, gender, place of residence, saturated fatty acids, and smoking, traditional DP was associated with higher risk of both diabetes (OR 2.00; 95% CI 1.33–3.02; *p* < 0.05) and IFG (OR 1.26; 95% CI 1.00–1.58; *p*< 0.05) prevalence. However, it was observed that the unhealthy DP was associated with a lower risk of diabetes prevalence (Table 5).

In a logistic regression model including age, BMI, energy intake, fiber, gender, place of residence, saturated fatty acids, and smoking, a lower risk for visceral obesity prevalence was observed for healthy DP (OR 0.68; 95% CI; 0.49–0.94; *p* < 0.05). In similar models, higher risk for being overweight and obesity prevalence was observed for unhealthy DP (OR 1.26; 95% CI 1.03–1.54, *p* < 0.05) and traditional DP (OR 1.59; 95% CI 1.24–2.03; *p* < 0.05) (Table 6). However, the unhealthy DP as a linear predictor of the risk for overweight and obesity prevalence was lower (OR 0.72; 95% CI 0.56–0.93). The negative coefficient of unhealthy DP (L) means that there is a general trend of decreasing the overweight and obesity prevalence with quartiles of the dietary pattern. However, the positive coefficient of unhealthy DP (Q) suggests that the trend is deaccelerated at the upper quartiles. Detailed results for the whole averaged models identified with the AIC are presented in Supplementary Materials (Tables S8–S13).

## 4. Discussion

In the herein study conducted among PURE Poland study participants, men and women living in urban and rural areas of Lower Silesia, three dietary patterns were identified using statistical methods. Subsequently, the association between these three DP and the prevalence of selected noncommunicable diseases, such as excessive body weight and visceral obesity, diabetes and impaired fasting glucose, hypertension, and total cardiovascular diseases, was assessed. Dietary patterns were named: healthy, unhealthy, and traditional, taking into account the dominating food groups in each of them (Table 1). In the healthy DP these were fruits, vegetables, nuts, seeds, raisins, and unrefined grains. In the unhealthy DP these groups were refined grains, sugar and honey, high fat cheese and cream, margarines and mayonnaise, and sweets. In the traditional DP, the dominating food groups were typical Polish mixed dishes and soups, red and processed meat, fish, and poultry.

Food groups characteristic for healthy DP were compatible with the European Society of Cardiology (ESC) recommendations for cardiovascular disease prevention [23]. The ESC defines a healthy diet as one that should contain more plant and less animal foods. It should include sources of polyunsaturated and monounsaturated fatty acids, dietary fiber, unsalted nuts (30 g per day), fruits (≥2–3 servings per day), vegetables (≥2–3 servings per day), and fish (1–2 times per week, in particular fatty fish). Among participants with the highest adherence to the healthy DP in comparison to the lowest adherence, there was significantly less people with hypertension, diabetes, IFG, and visceral obesity (Table 2). Moreover, in the logistic regression model including gender, place of residence, age, BMI, energy intake, dietary fiber and saturated fatty acids intake, and smoking, the decreased risk of visceral obesity was observed with increased the quartiles of healthy DP (Table 6). These findings are consistent with the results of other studies, where the diets similar to the Mediterranean diet contributed to a significant improvement in the health status, which was associated with a significant reduction in the all-cause mortality (9%), as well as the reduction in cardiovascular and cancer mortality (9% and 6%, respectively) and prevalence of Parkinson’s and Alzheimer’s disease (13%) [24]. These findings seem to be clinically significant for public health; in particular they justify the use of the Mediterranean diet and diets similar to it as a pattern in the primary prevention of chronic diseases such as CVD, diabetes [25], obesity [26], visceral obesity [27], and hypertension [28]. The DASH diet, in turn, proved to be effective in lowering hypertension in a group of hypertensive patients [28,29]. Results of the PREDIMED study (PREvención con DIeta MEDiterránea), that involved 7 447 participants at high cardiovascular risk observed for an average of 5 years, confirmed that the Mediterranean diet supplemented with extra virgin olive oil or nuts reduced the prevalence of cardiovascular events by 30%. This protective effect of the Mediterranean diet has been attributed to its impact on insulin sensitivity, blood pressure, lipid profile, oxidative stress, and inflammatory biomarkers associated with atherosclerosis. These effects are independent of gene polymorphisms associated with lipid changes or the inflammatory response, which confirms that the Mediterranean diet is a useful tool in the prevention of cardiovascular diseases and their main risk factors, including diabetes [30].

A healthy diet according to the ESC recommendations should reduce saturated and trans fatty acids, salt, red and processed meat, alcohol, and sugar-sweetened beverages [23]. The sources of animal fats and fast-digesting carbohydrates were characteristics for the dietary pattern named unhealthy in the herein study. In the herein study, there were significantly more participants with IFG in the fourth quartile of the unhealthy dietary pattern in comparison to the first quartile. The similar trend was observed in the study conducted among 8752 adult Indonesian Population who were overweight or obese, where the intake of sweet, grilled, and processed foods was associated with IFG. Moreover, it was also found that impaired glucose tolerance (IGT) was associated with the intake of high-fat foods [31]. Similar results were observed in the study conducted among 7555 Chinese adults, where the association between dietary patterns and prediabetes was analyzed. It was found that dietary patterns abundant in meat, but poor in fresh fruits and vegetables, as well as milk and fish were positively associated with the higher risk of prediabetes, especially the risk of IFG. On the other hand, the dietary pattern called healthy diet, which mainly included fresh fruits, vegetables, milk, and eggs, was associated with a lower risk of prediabetes [32]. Trend analysis in our study showed that there was no association between diabetes prevalence and unhealthy DP. However, unhealthy DP was associated a lower risk of diabetes in the logistic regression model including gender, place of residence, age, energy intake, dietary fiber, saturated fatty acids intake, and smoking. The justification for this result is hard to explain based on our available data. However, we suppose that this could be related to the participants’ awareness about the presence of diabetes among them. In the fourth quartile of unhealthy DP, among 45 participants with diabetes, 64% of them knew about the disease, while in the first quartile among 66 participants with diabetes, 79% knew about the disease. This means that in the quartile with the highest adherence to the unhealthy DP, there was a lower percentage of diabetic participants who knew about their disease (the disease was diagnosed earlier before the study, the data were taken from medical history) in comparison to the quartile with the lowest adherence to the unhealthy DP. Probably the greater awareness of the recommended diet in diabetes caused the intentional elimination from the diet of products which where characteristic for unhealthy DP in this study (refined grains, sweets, sugar, and honey).

In the present study, a negative trend between higher adherence to the unhealthy dietary pattern and the prevalence of hypertension was observed. But also in the logistic regression model including gender, place of residence, age, BMI, energy intake, dietary fiber, saturated fatty acids intake, and smoking, the unhealthy DP was associated with a lower risk of hypertension. It is likely that this observation is due to the association between hypertension and obesity, as it was shown in the same group of people that participants with obesity had 2.5-fold higher odds for hypertension. Moreover, obesity was the third predictor of hypertension prevalence after age and male gender [33]. In the herein study the logistic regression model showed a lower risk of excessive body weight for unhealthy DP as a linear predictor but a positive coefficient of unhealthy DP (quadratic) suggests that the trend is deaccelerated at upper quartiles.

In the present study, the traditional DP—which mainly included typical Polish mixed dishes with a high content of saturated fatty acids as well as red and processed meat and soups, but with low content of fruits, nuts, and seeds (Table 4)—was positively associated with the prevalence of total CVD, diabetes, IFG, visceral obesity, being overweight, and obesity. In the logistic regression model including gender, place of residence, age, energy intake, dietary fiber and saturated fatty acids intake, and smoking, the traditional DP was associated with a higher risk of diabetes and IFG (Table 5), being overweight and obesity (Table 6). The least favorable in terms of cardiovascular prevention in this study was traditional DP, abundant in foods being sources of saturated fatty acids, such as red and processed meat, poultry, and fish, but poor in plant foods. Low intake of compounds occurring in plants (dietary fiber, minerals, vitamins, flavonoids) and excessive intake of animal fats are known predictors of CVD [2,3].

Taking into account the results obtained in this study, an important achievement was identifying dietary patterns which include dishes characteristic of Polish cuisine, and what is worth noting, this DP was negatively associated with the prevalence of noncommunicable diseases in this study. Therefore, from the point of view of health prevention, the Polish population should be encouraged to implement modification into typical recipes of Polish dishes focused on the limitation of animal fats and increased plant oils (canola oil or olive oil instead of lard or butter), limitation of red and processed meat (poultry instead of pork, fresh meat instead of processed meat), increasing whole grain (whole grain or rye flour instead of wheat flour), limitation of fried breaded food (cooking or stewed instead of frying), increased fresh vegetables, fruits, and nuts. These simple modifications will help to improve diet quality and thereby improve health status. It would be helpful to increase and facilitate access to dieticians in public healthcare institutions and hospitals, where people could receive nutritional education.

Our study has some limitations. First of all, data was collected on the baseline of the PURE study in the years 2007–2009. However, new data are still collected during follow-up, but there were not enough new incidents to assess the relation between them and diet. Therefore, the study will be updated with new data in the future. Secondly, we used BMI as an indicative of excessive body weight, which does not take into account the body composition. However, in this study we also used data about waist circumference, which indicates the presence of visceral obesity. Moreover ESC [20] emphasized that adiposity is an important risk factor, which increases CVD risk, and showed a meta-analysis conclusion that both BMI and waist circumference are similarly, strongly, and continuously associated with atherosclerotic cardiovascular disease and type 2 diabetes. Another limitation might be the use of self-report data. On the other hand, the study is not based only on these data, but also included biochemical test results collected during the study. Moreover, the dietary data were collected retrospectively, which can be subject to memory bias. However, most of the epidemiological studies are based on different retrospective dietary recalls methods.

## 5. Conclusions

The dietary pattern abundant in fruits, vegetables, seeds, nuts, raisins, and unrefined grains, named “healthy dietary pattern”, had a beneficial association with a lower prevalence of selected noncommunicable diseases. Dietary patterns abundant in meat products, but poor in fruits and vegetables were positively associated with a higher prevalence of total CVD, diabetes, IFG, excessive body weight, and visceral obesity.

## Figures and Tables

**Table 1:** Factor loadings matrix for dietary patterns identified in the study group.

Food Group	Unhealthy Dietary Pattern	Healthy Dietary Pattern	Traditional Dietary Pattern
Milk low fat	0.20	0.45	0.05
High fat cheese, cream	**0.53**	0.28	0.12
Margarines and mayonnaise	**0.53**	−0.11	0.06
Animal fats	0.39	0.04	0.11
Eggs	0.41	−0.06	0.15
Fish	0.13	0.14	**0.55**
Unrefined grains	−0.16	**0.50**	0.18
Refined grains	**0.61**	−0.30	0.22
Mixed dishes	0.08	0.13	**0.74**
Soups	−0.03	0.15	**0.71**
Alcohol	0.01	−0.15	0.17
Sweets	**0.52**	0.28	0.23
Beverages	0.03	0.16	−0.11
Sugar and honey	**0.54**	0.03	−0.03
Nuts, seeds, raisins	0.00	**0.61**	0.03
Fruits	0.01	**0.68**	0.05
Juices	0.27	0.30	0.21
Vegetables	−0.04	**0.61**	0.42
Tea, coffee	0.35	0.22	−0.17
Red and processed meat	0.45	−0.09	**0.64**
Poultry	0.29	0.15	**0.51**
Potatoes and chips	0.24	−0.05	0.34
%	0.11	0.10	0.12

Factor loadings ≥ 0.5 were bolded.

**Table 2:** Prevalence of selected noncommunicable diseases between quartiles of Healthy Dietary Pattern.

Incidents	Healthy Dietary Pattern				
	Q1 *n* (%)	Q2 *n* (%)	Q3 *n* (%)	Q4 *n* (%)	*p* Trend
CVD total (*n*: Q1 = 505, Q2 = 506, Q3 = 506, Q4 = 506)	68 (13.5)	73 (14.4)	72 (14.2)	81 (16.0)	0.3490
Hypertension (*n*: Q1 = 502, Q2 = 498, Q3 = 502, Q4 = 504)	336 (66.9)	297 (59.6)	294 (58.6)	280 (55.6)	0.0003
Diabetes (*n*: Q1 = 464, Q2 = 474, Q3 = 473, Q4 = 478)	64 (13.8)	52 (11.0)	41 (8.7)	42 (8.8)	0.0009
IFG (*n*: Q1 = 464, Q2 = 474, Q3 = 473, Q4 = 478)	127 (27.4)	121 (25.5)	110 (23.3)	107 (22.4)	0.0089
Visceral obesity (*n*: Q1 = 505, Q2 = 505, Q3 = 506, Q4 = 506)	353 (69.9)	365 (72.3)	349 (69.0)	327 (64.6)	0.0348
Overweight + obesity (*n*: Q1 = 505, Q2 = 505, Q3 = 506, Q4 = 506)	365 (72.3)	364 (72.1)	358 (70.8)	350 (69.2)	0.2260

*n*—number of participants, CVD—cardiovascular diseases, IFG—Impaired fasting glucose, Q—quartile.

**Table 3:** Prevalence of selected noncommunicable diseases between quartiles of Unhealthy Dietary Pattern.

Incidents	Unhealthy Dietary Pattern				
	Q1 *n* (%)	Q2 *n* (%)	Q3 *n* (%)	Q4 *n* (%)	*p* Trend
CVD total (*n*: Q1 = 505, Q2 = 506, Q3 = 506, Q4 = 506)	81 (16.0)	75 (14.8)	64 (12.7)	74 (14.6)	0.3010
Hypertension (*n*: Q1 = 504, Q2 = 505, Q3 = 500, Q4 = 497)	338 (67.1)	302 (59.8)	279 (55.8)	288 (58.0)	0.0012
Diabetes (*n*: Q1 = 464, Q2 = 474, Q3 = 473, Q4 = 478)	66 (13.9)	41 (8.6)	47 (9.8)	45 (9.8)	0.1950
IFG (*n*: Q1 = 464, Q2 = 474, Q3 = 473, Q4 = 478)	108 (22.7)	95 (20.0)	121 (25.3)	141 (30.7)	0.0037
Visceral obesity (*n*: Q1 = 505, Q2 = 506, Q3 = 505, Q4 = 506)	356 (70.5)	346 (68.4)	324 (64.2)	368 (72.7)	0.8120
Overweight + obesity (*n*: Q1 = 505, Q2 = 506, Q3 = 505, Q4 = 506)	381 (75.5)	365 (72.1)	325 (64.4)	366 (72.3)	0.0542

*n*—number of participants, CVD—cardiovascular diseases, IFG—Impaired fasting glucose, Q—quartile.

**Table 4:** Prevalence of selected noncommunicable diseases between quartiles of Traditional Dietary Pattern.

Incidents	Traditional Dietary Pattern				
	Q1 *n* (%)	Q2 *n* (%)	Q3 *n* (%)	Q4 *n* (%)	*p* Trend
CVD total (*n*: Q1 = 505, Q2 = 506, Q3 = 506, Q4 = 506)	54 (10.7)	80 (15.8)	80 (15.8)	80 (15.8)	0.0391
Hypertension (*n*: Q1 = 495, Q2 = 502, Q3 = 505, Q4 = 504)	285 (57.6)	297 (59.2)	310 (61.4)	315 (62.5)	0.0905
Diabetes (*n*: Q1 = 464, Q2 = 474, Q3 = 473, Q4 = 478)	28 (5.9)	49 (10.3)	61 (13.0)	61 (13.1)	0.0014
IFG (*n*: Q1 = 464, Q2 = 474, Q3 = 473, Q4 = 478)	107 (22.3)	106 (22.4)	118 (25.1)	134 (28.8)	0.0000
Visceral obesity (*n*: Q1 = 505, Q2 = 506, Q3 = 505, Q4 = 506)	317 (62.8)	344 (68.0)	367 (72.7)	366 (72.3)	0.0003
Overweight + obesity (*n*: Q1 = 505, Q2 = 506, Q3 = 505, Q4 = 506)	325 (64.4)	347 (68.6)	380 (75.3)	385 (76.1)	0.0000

*n*—number of participants, CVD—cardiovascular diseases, IFG—Impaired fasting glucose, Q—quartile.

**Table 5:** OR calculated using averaged models for CVD, hypertension, diabetes, and IFG prevalence in relation to dietary patterns adjusted for other predictors (see explanations below the table).

Model	Predictors	OR (95% CI)	*p*
CVD total prevalence vs.			
Healthy Dietary Pattern ^1)^	Healthy DP (L)	1.33 (0.98–1.79)	0.0654
	Healthy DP (C)	0.93 (0.71–1.22)	0.6009
	Healthy DP (Q)	1.06 (0.81–1.38)	0.6916
Unhealthy Dietary Pattern ^1)^	Unhealthy DP (L)	0.75 (0.55–1.03)	0.0747
	Unhealthy DP (C)	1.11 (0.85–1.46)	0.4273
	Unhealthy DP (Q)	1.06 (0.81–1.38)	0.6720
Traditional Dietary Pattern ^1)^	Traditional DP (L)	1.17 (0.86–1.61)	0.3204
	Traditional DP (C)	1.11 (0.86–1.44)	0.41531
	Traditional DP (Q)	0.88 (0.67–1.16)	0.36456
Hypertension prevalence vs.			
Healthy Dietary Pattern ^2)^	Healthy DP (L)	0.79 (0.62–1.01)	0.0611
	Healthy DP (C)	0.84 (0.69–1.02)	0.0864
	Healthy DP (Q)	1.03 (0.84–1.26)	0.7852
Unhealthy Dietary Pattern ^2)^	Unhealthy DP (L)	0.77 (0.62–0.95)	0.01550
	Unhealthy DP (C)	0.96 (0.79–1.17)	0.70046
	Unhealthy DP (Q)	1.11 (0.91–1.36)	0.29305
Traditional Dietary Pattern ^2)^	Traditional DP (L)	0.96 (0.77–1.19)	0.71454
	Traditional DP (C)	1.00 (0.82–1.22)	0.99471
	Traditional DP (Q)	1.06 (0.87–1.30)	0.54251
Diabetes prevalence vs.			
Healthy Dietary Pattern ^1)^	Healthy DP (L)	0.72 (0.48–1.07)	0.10792
	Healthy DP (C)	1.00 (0.72–1.39)	0.98100
	Healthy DP (Q)	1.19 (0.86–1.66)	0.29413
Unhealthy Dietary Pattern ^1)^	Unhealthy DP (L)	0.47 (0.31–0.70)	0.00026
	Unhealthy DP (C)	0.80 (0.57–1.11)	0.17884
	Unhealthy DP (Q)	1.34 (0.96–1.85)	0.08238
Traditional Dietary Pattern ^1)^	Traditional DP (L)	2.00 (1.33–3.02)	0.00093
	Traditional DP (C)	1.05 (0.77–1.44)	0.74857
	Traditional DP (Q)	1.00 (0.71–1.41)	0.99898
Impaired fasting glucose (IFG) prevalence			
Healthy Dietary Pattern ^1)^	Healthy DP (L)	0.96 (0.76–1.22)	0.74915
	Healthy DP (C)	1.01 (0.81–1.26)	0.94157
	Healthy DP (Q)	1.09 (0.87–1.36)	0.46593
Unhealthy Dietary Pattern ^1)^	Unhealthy DP (L)	0.97 (0.75–1.26)	0.82719
	Unhealthy DP (C)	0.86 (0.69–1.08)	0.18673
	Unhealthy DP (Q)	1.19 (0.95–1.49)	0.12158
Traditional Dietary Pattern ^1)^	Traditional DP (L)	1.14 (0.90–1.44)	0.28332
	Traditional DP (C)	0.99 (0.79–1.24)	0.91353
	Traditional DP (Q)	1.26 (1.00–1.58)	0.04730

L, Q, C—the coefficients of linear, quadratic, and cubic relationships, respectively other variables in models: ^1)^ age, energy intake, fiber, gender, place of residence, saturated fatty acids, smoking, ^2)^ age, BMI, energy intake, fiber, gender, place of residence, saturated fatty acids, smoking.

**Table 6:** OR calculated using averaged models for visceral obesity, overweight, and obesity prevalence in relation to dietary patterns adjusted for other predictors (see explanations below the table).

Model	Predictors	OR (95% CI)	*p*
Visceral obesity prevalence vs.			
Healthy Dietary Pattern ^1)^	Healthy DP (L)	0.68 (0.49–0.94)	0.02100
	Healthy DP (C)	1.11 (0.83–1.48)	0.48976
	Healthy DP (Q)	0.83 (0.62–1.10)	0.19463
Unhealthy Dietary Pattern ^1)^	Unhealthy DP (L)	1.44 (0.97–2.12)	0.06949
	Unhealthy DP (C)	1.08 (0.82–1.44)	0.57688
	Unhealthy DP (Q)	1.02 (0.76–1.37)	0.88244
Traditional Dietary Pattern ^1)^	Traditional DP (L)	0.90 (0.66–1.23)	0.52298
	Traditional DP (C)	1.04 (0.78–1.40)	0.77353
	Traditional DP (Q)	1.07 (0.80–1.43)	0.63778
Overweight and obesity prevalence vs.			
Healthy Dietary Pattern ^1)^	Healthy DP (L)	1.06 (0.83–1.36)	0.6361
	Healthy DP (C)	0.96 (0.78–1.17)	0.6581
	Healthy DP (Q)	0.91 (0.74–1.12)	0.3766
Unhealthy Dietary Pattern ^1)^	Unhealthy DP (L)	0.72 (0.56–0.93)	0.01114
	Unhealthy DP (C)	1.20 (0.98–1.46)	0.07436
	Unhealthy DP (Q)	1.26 (1.03–1.54)	0.02613
Traditional Dietary Pattern ^1)^	Traditional DP (L)	1.59 (1.24–2.03)	0.00022
	Traditional DP (C)	0.88 (0.72–1.08)	0.20943
	Traditional DP (Q)	0.96 (0.78–1.18)	0.72838

L, Q, C—the coefficients of linear, quadratic, and cubic relationships, respectively other variables in models: ^1)^ age, BMI, energy intake, fiber, gender, place of residence, saturated fatty acids, smoking.

## Data Availability

Not applicable.

## Supplementary Materials

The following supporting information can be downloaded at https://www.mdpi.com/article/10.3390/nu15163524/s1, Table S1: The average daily intake of each food group taking into account in the principal components analysis (PCA) performed for the identification of dietary patterns; Table S2: The best models built using best predictor subset selection mode (according AIC) for CVD total prevalence and different dietary patterns; Table S3: The best models built using best predictor subset selection mode (according AIC) for diabetes prevalence and different dietary patterns; Table S4: The best models built using best predictor subset selection mode (according AIC) for IFG prevalence and different dietary patterns; Table S5: The best models built using best predictor subset selection mode (according AIC) for hypertension prevalence and different dietary patterns; Table S6: The best models built using best predictor subset selection mode (according AIC) for overweight and obesity prevalence and different dietary patterns; Table S7: The best models built using best predictor subset selection mode (according AIC) for visceral obesity prevalence and different dietary patterns; Table S8: OR calculated using averaged models for CVD prevalence in relation to dietary patterns adjusted for other predictors; Table S9: OR calculated using averaged models for hypertension prevalence in relation to dietary patterns adjusted for other predictors; Table S10: OR calculated using averaged models for diabetes prevalence in relation to dietary patterns adjusted for other predictors; Table S11: OR calculated using averaged models for impaired fasting glucose prevalence in relation to dietary patterns adjusted for other predictors; Table S12: OR calculated using averaged models for visceral obesity prevalence in relation to dietary patterns adjusted for other predictors; Table S13: OR calculated using averaged models for overweight and obesity prevalence in relation to dietary patterns adjusted for other predictors.
