# Supplementary material for: Dietary Patterns and the Prevalence of Noncommunicable Diseases in the PURE Poland Study Participants
Source: Nutrients. 2023 Aug 10;15(16):3524. doi: 10.3390/nu15163524 (PMC10459498; doi:10.3390/nu15163524)
Supplement: Supplementary file 1 [file nutrients-15-03524-s001.zip › nutrients-2493737-supplementary.pdf]

Table S1. The average daily intake of each food group taking into account in the principal components analysis (PCA) performed for the identification of dietary patterns

**Food group**	**Intake [g/day]**
Milk low fat	223.6 ± 220.1
High fat cheese, cream	28.8 ± 23.0
Margarines and mayonnaise	6.7 ± 6.6
Animal fats	13.9 ± 15.8
Eggs	15.6 ± 15.1
Fish	13.9 ± 11.1
Unrefined grains	87.8 ± 85.8
Refined grains	77.8 ± 65.0
Mixed dishes	33.4 ± 22.4
Soups	249.8 ± 144.8
Alcohol	56.9 ± 127.5
Sweets	48.5 ± 38.2
Beverages	199.6 ± 342.2
Sugar and honey	17.0 ± 16.1
Nuts, seeds, raisins	14.3 ± 22.3
Fruits	296.7 ± 212.1
Juices	133.8 ± 141.7
Vegetables	312.8 ± 195.2
Tea, coffee	943.5 ± 467.9
Red and processed meat	75.5 ± 47.0
Poultry	52.9 ± 37.4
Potatoes and chips	90.4 ± 59.2

Table S2. The best models built using best predictor subset selection mode (according AIC) for CVD total prevalence and different dietary patterns

**(Intercept)**	**Age**	**BMI**	**Healthy DP**	**Energy intake**	**Fiber**	**Gender**	**Physical activity**	**Place of residence**	**SFA**	**Smoking**	**R^2**	**adjR^2**	**df**	**AIC**	**Weight**
-6.97	0.099		+					+	-0.03	+	0.096	0.17	9	1490.06	0.19
-7.32	0.098		+					+		+	0.095	0.17	8	1490.14	0.18
-7.24	0.098		+	-0.0003	0.016			+		+	0.097	0.17	10	1490.26	0.17
-7.48	0.098		+		0.004			+		+	0.096	0.17	9	1491.60	0.09
-7.15	0.098		+	-7.51194E-05				+		+	0.096	0.17	9	1491.62	0.09
-6.87	0.099		+	-5.25222E-05				+	-0.03	+	0.097	0.17	10	1491.81	0.08
-7.09	0.099		+		0.003			+	-0.03	+	0.096	0.17	10	1491.89	0.07
-7.12	0.099		+	-0.0002	0.014			+	-0.01	+	0.097	0.17	11	1492.04	0.07
-6.97	0.099		+			+		+	-0.03	+	0.096	0.17	10	1492.06	0.07
**(Intercept)**	**Age**	**BMI**	**Unhealthy DP**	**Energy intake**	**Fiber**	**Gender**	**Physical activity**	**Place of residence**	**SFA**	**Smoking**	**R^2**	**adjR^2**	**df**	**AIC**	**Weight**
-7.62	0.098		+		0.011			+		+	0.097	0.17	9	1489.00	0.39
-7.53	0.098		+	-0.0001	0.015			+		+	0.097	0.17	10	1490.70	0.16
-7.49	0.098		+		0.010			+	-0.01	+	0.097	0.17	10	1490.78	0.16
-7.62	0.098		+		0.011	+		+		+	0.097	0.17	10	1490.91	0.15
-7.25	0.097		+					+		+	0.095	0.17	8	1490.99	0.14
**(Intercept)**	**Age**	**BMI**	**Traditional DP**	**Energy intake**	**Fiber**	**Gender**	**Physical activity**	**Place of residence**	**SFA**	**Smoking**	**R^2**	**adjR^2**	**df**	**AIC**	**Weight**
-7.32	0.098		+	-0.0003	0.019			+		+	0.097	0.17	10	1490.94	0.30
-7.34	0.097		+					+		+	0.095	0.17	8	1492.06	0.17
-7.00	0.098		+					+	-0.03	+	0.096	0.17	9	1492.07	0.17
-7.59	0.097		+		0.008			+		+	0.095	0.17	9	1492.49	0.14
-7.24	0.098		+	-0.0003	0.018			+	-0.01	+	0.097	0.17	11	1492.84	0.12
-7.31	0.098		+	-0.0003	0.019	+		+		+	0.097	0.17	11	1492.89	0.11

CVD – cardiovascular disease; BMI – body mass index; DP – dietary pattern; SFA – saturated fatty acids; AIC - Akaike information criterion

Table S3. The best models built using best predictor subset selection mode (according AIC) for diabetes prevalence and different dietary patterns

**(Intercept)**	**Age**	**Healthy DP**	**Energy intake**	**Fiber**	**Gender**	**Physical activity**	**Place of residence**	**SFA**	**Smoking**	**R^2**	**adjR^2**	**df**	**AIC**	**Weight**
-4.39	0.056	+	-0.0007	0.04	+		+		+	0.097	0.17	11	1029.32	0.24
-4.40	0.056	+	-0.0006	0.04			+		+	0.095	0.17	10	1029.45	0.23
-4.78	0.055	+	-0.0008	0.05	+		+	0.03	+	0.098	0.18	12	1029.89	0.18
-4.74	0.056	+	-0.0007	0.05			+	0.03	+	0.096	0.17	11	1030.36	0.14
-4.21	0.055	+	-0.0007	0.04	+		+			0.093	0.17	9	1030.70	0.12
-4.59	0.054	+	-0.0008	0.05	+		+	0.03		0.094	0.17	10	1031.32	0.09
**(Intercept)**	**Age**	**Unhealthy DP**	**Energy intake**	**Fiber**	**Gender**	**Physical activity**	**Place of residence**	**SFA**	**Smoking**	**R^2**	**adjR^2**	**df**	**AIC**	**Weight**
-4.83	0.056	+		0.015	+		+		+	0.103	0.18	10	1017.89	0.28
-4.76	0.056	+		0.014			+		+	0.101	0.18	9	1018.20	0.24
-4.63	0.056	+	-0.0002	0.024	+		+		+	0.103	0.19	11	1019.10	0.15
-4.61	0.055	+		0.015	+		+			0.099	0.18	8	1019.75	0.11
-4.96	0.056	+		0.016	+		+	0.01	+	0.103	0.19	11	1019.76	0.11
-4.61	0.056	+	-0.0002	0.021			+		+	0.101	0.18	10	1019.80	0.11
**(Intercept)**	**Age**	**Traditional DP**	**Energy intake**	**Fiber**	**Gender**	**Physical activity**	**Place of residence**	**SFA**	**Smoking**	**R^2**	**adjR^2**	**df**	**AIC**	**Weight**
-3.75	0.055	+	-0.0009	0.035	+		+		+	0.101	0.18	11	1021.67	0.35
-3.68	0.056	+	-0.0008	0.032			+		+	0.100	0.18	10	1022.07	0.29
-3.99	0.055	+	-0.0009	0.040	+		+	0.02	+	0.102	0.18	12	1023.04	0.18
-3.54	0.055	+	-0.0009	0.034	+		+			0.098	0.18	9	1023.05	0.18

CVD – cardiovascular disease; BMI – body mass index; DP – dietary pattern; SFA – saturated fatty acids; AIC - Akaike information criterion

Table S4. The best models built using best predictor subset selection mode (according AIC) for IFG prevalence and different dietary patterns

**(Intercept)**	**Age**	**Healthy DP**	**Energy intake_kod**	**Fiber**	**Gender**	**Physical activity**	**Place of residence**	**SFA**	**Smoking**	**R^2**	**adjR^2**	**df**	**AIC**	**Weight**
-1.68	0.021	+					+		+	0.061	0.09	8	1897.77	0.34
-1.77	0.021	+	+				+		+	0.062	0.09	9	1898.58	0.23
-1.79	0.021	+		0.003			+		+	0.061	0.09	9	1899.36	0.15
-1.54	0.021	+					+	-0.010	+	0.061	0.09	9	1899.39	0.15
-1.68	0.021	+			+		+		+	0.061	0.09	9	1899.77	0.13
**(Intercept)**	**Age**	**Unhealthy DP**	**Energy intake_kod**	**Fiber**	**Gender**	**Physical activity**	**Place of residence**	**SFA**	**Smoking**	**R^2**	**adjR^2**	**df**	**AIC**	**Weight**
-1.67	0.021	+					+		+	0.063	0.09	8	1894.32	0.35
-1.74	0.021	+	+				+		+	0.064	0.09	9	1895.18	0.23
-1.53	0.021	+					+	-0.011	+	0.063	0.09	9	1895.88	0.16
-1.73	0.021	+		0.002			+		+	0.063	0.09	9	1896.13	0.14
-1.67	0.021	+			+		+		+	0.063	0.09	9	1896.32	0.13
**(Intercept)**	**Age**	**Traditional DP**	**Energy intake_kod**	**Fiber**	**Gender**	**Physical activity**	**Place of residence**	**SFA**	**Smoking**	**R^2**	**adjR^2**	**df**	**AIC**	**Weight**
-1.69	0.022	+					+		+	0.064	0.09	8	1892.92	0.38
-1.56	0.022	+					+	-0.010	+	0.064	0.09	9	1894.55	0.17
-1.73	0.022	+	+				+		+	0.064	0.09	9	1894.67	0.16
-1.64	0.022	+		-0.002			+		+	0.064	0.09	9	1894.81	0.15
-1.69	0.022	+			+		+		+	0.064	0.09	9	1894.92	0.14

CVD – cardiovascular disease; BMI – body mass index; DP – dietary pattern; SFA – saturated fatty acids; AIC - Akaike information criterion; Energy intake_kod – categorical variable by median value

Table S5. The best models built using best predictor subset selection mode (according AIC) for hypertension prevalence and different dietary patterns

(Intercept)	Age	BMI_4	Healthy DP	Energy intake	Fiber	Gender	Physical activity	Place of residence	SFA	Smoking	R^2	adjR^2	df	AIC	Weight
-3.99	0.057	+	+		0.007	+		+			0.164	0.22	11	2358.91	0.12
-3.83	0.057	+	+	-0.0002	0.014	+		+			0.165	0.22	12	2359.03	0.11
-3.75	0.057	+	+			+		+			0.164	0.22	10	2359.18	0.11
-3.87	0.056	+	+		0.007	+		+		+	0.166	0.22	13	2359.19	0.10
-3.64	0.056	+	+			+		+		+	0.165	0.22	12	2359.43	0.09
-3.73	0.056	+	+	-0.0002	0.014	+		+		+	0.167	0.23	14	2359.56	0.09
-3.54	0.056	+	+	-0.0002	0.016	+				+	0.166	0.22	13	2359.95	0.07
-3.62	0.057	+	+	-0.0002	0.017	+					0.164	0.22	11	2360.01	0.07
-3.54	0.057	+	+			+		+	-0.016		0.164	0.22	11	2360.07	0.07
-3.81	0.057	+	+		0.006	+		+	-0.011		0.165	0.22	12	2360.36	0.06
-3.43	0.056	+	+			+		+	-0.015	+	0.166	0.22	13	2360.39	0.06
-3.70	0.056	+	+		0.006	+		+	-0.011	+	0.166	0.22	14	2360.69	0.05
**(Intercept)**	**Age**	**BMI_4**	**Unhealthy DP**	**Energy intake**	**Fiber**	**Gender**	**Physical activity**	**Place of residence**	**SFA**	**Smoking**	**R^2**	**adjR^2**	**df**	**AIC**	**Weight**
-3.53	0.056	+	+			+					0.163	0.22	9	2357.76	0.22
-3.44	0.055	+	+			+				+	0.165	0.22	11	2358.47	0.15
-3.61	0.056	+	+			+		+			0.164	0.22	10	2358.64	0.14
-3.65	0.056	+	+		0.004	+					0.164	0.22	10	2358.82	0.13
-3.39	0.056	+	+			+			-0.011		0.164	0.22	10	2359.18	0.11
-3.64	0.056	+	+	5.39E-05		+					0.163	0.22	10	2359.35	0.10
-3.50	0.055	+	+			+		+		+	0.165	0.22	12	2359.71	0.08
-3.55	0.055	+	+		0.003	+				+	0.165	0.22	12	2359.74	0.08
**(Intercept)**	**Age**	**BMI_4**	**Traditional DP**	**Energy intake**	**Fiber**	**Gender**	**Physical activity**	**Place of residence**	**SFA**	**Smoking**	**R^2**	**adjR^2**	**df**	**AIC**	**Weight**
-3.69	0.056	+	+			+		+			0.161	0.22	10	2364.43	0.12
-3.51	0.056	+	+	-0.0002	0.012	+					0.162	0.22	11	2364.88	0.10
-3.45	0.056	+	+			+		+	-0.018		0.162	0.22	11	2364.99	0.09
-3.32	0.057	+	+			+			-0.021		0.161	0.22	10	2365.14	0.09
-3.58	0.056	+	+			+		+		+	0.163	0.22	12	2365.27	0.08
-3.59	0.057	+	+			+					0.160	0.22	9	2365.31	0.08
-3.49	0.056	+	+			+				+	0.162	0.22	11	2365.31	0.08
-3.23	0.056	+	+			+			-0.020	+	0.163	0.22	12	2365.35	0.08
-3.41	0.055	+	+	-0.0002	0.011	+				+	0.163	0.22	13	2365.64	0.07
-3.59	0.056	+	+	-0.0002	0.010	+		+			0.162	0.22	12	2365.83	0.06
-3.35	0.055	+	+			+		+	-0.017	+	0.163	0.22	13	2365.88	0.06
-3.58	0.056	+	+	-4.8E-05		+		+			0.161	0.22	11	2366.08	0.05
-3.77	0.056	+	+		0.003	+		+			0.161	0.22	11	2366.10	0.05

CVD – cardiovascular disease; BMI – body mass index; DP – dietary pattern; SFA – saturated fatty acids; AIC - Akaike information criterion; BMI_4 – categorical variable: underweight <18,5 kg/m^2, normal body weight 18,5-24,9 kg/m^2; overweight 25-29,9 kg/m^2; obesity ≥30 kg/m^2

Table S6. The best models built using best predictor subset selection mode (according AIC) for overweight and obesity prevalence and different dietary patterns

**(Intercept)**	**Age_kod**	**Healthy DP**	**Energy intake**	**Fiber**	**Gender**	**Physical activity**	**Place of residence**	**SFA**	**Smoking**	**R^2**	**adjR^2**	**df**	**AIC**	**Weight**
0.45	+	+	-0.0002	0.016	+		+		+	0.068	0.10	11	2311.54	0.40
0.47	+	+			+		+		+	0.066	0.09	9	2312.57	0.24
0.27	+	+		0.006	+		+		+	0.067	0.10	10	2312.94	0.20
0.37	+	+	-0.0002	0.017	+		+	0.006	+	0.068	0.10	12	2313.41	0.16
**(Intercept)**	**Age_kod**	**Unhealthy DP**	**Energy intake**	**Fiber**	**Gender**	**Physical activity**	**Place of residence**	**SFA**	**Smoking**	**R^2**	**adjR^2**	**df**	**AIC**	**Weight**
0.29	+	+		0.008	+		+		+	0.074	0.11	10	2296.35	0.37
0.27	+	+	0.0001		+		+		+	0.074	0.11	10	2297.83	0.18
0.53	+	+			+		+		+	0.073	0.10	9	2297.92	0.17
0.18	+	+		0.008	+		+	0.007	+	0.074	0.11	11	2298.14	0.15
0.32	+	+	-4.5E-05	0.010	+		+		+	0.074	0.11	11	2298.27	0.14
**(Intercept)**	**Age_kod**	**Traditional DP**	**Energy intake**	**Fiber**	**Gender**	**Physical activity**	**Place of residence**	**SFA**	**Smoking**	**R^2**	**adjR^2**	**df**	**AIC**	**Weight**
0.74	+	+	-0.0003	0.012	+		+		+	0.075	0.11	11	2297.26	0.49
0.77	+	+	-0.0002		+		+		+	0.074	0.11	10	2298.14	0.32
0.65	+	+	-0.0003	0.013	+		+	0.007	+	0.075	0.11	12	2299.10	0.20

CVD – cardiovascular disease; BMI – body mass index; DP – dietary pattern; SFA – saturated fatty acids; AIC - Akaike information criterion; Age_kod – categorical variable by median value

Table S7. The best models built using best predictor subset selection mode (according AIC) for visceral obesity prevalence and different dietary patterns

(Intercept)	Age	BMI	Healthy DP	Energy intake	Fiber	Gender	Physical activity	Place of residence	SFA	Smoking	R^2	adjR^2	df	AIC	Weight
-19.09	0.030	0.75	+			+		+			0.480	0.68	8	1200.69	0.29
-19.36	0.031	0.75	+			+		+		+	0.481	0.68	10	1201.29	0.22
-19.33	0.031	0.75	+	8.8E-05		+		+			0.480	0.68	9	1202.07	0.15
-19.23	0.030	0.75	+		0.004	+		+			0.480	0.68	9	1202.30	0.13
-19.17	0.030	0.75	+			+		+	0.006		0.480	0.68	9	1202.63	0.11
-19.61	0.032	0.75	+	8.89E-05		+		+		+	0.481	0.68	11	1202.65	0.11
**(Intercept)**	**Age**	**BMI**	**Unhealthy DP**	**Energy intake**	**Fiber**	**Gender**	**Physical activity**	**Place of residence**	**SFA**	**Smoking**	**R^2**	**adjR^2**	**df**	**AIC**	**Weight**
-18.78	0.031	0.75	+	-0.0002		+		+			0.479	0.67	9	1204.91	0.20
-19.08	0.032	0.75	+	-0.0002		+		+		+	0.480	0.68	11	1205.40	0.16
-19.10	0.031	0.75	+			+		+			0.478	0.67	8	1205.45	0.15
-19.41	0.032	0.75	+			+		+		+	0.479	0.67	10	1205.56	0.14
-18.96	0.031	0.75	+		-0.007	+		+			0.479	0.67	9	1206.07	0.11
-19.26	0.032	0.75	+		-0.006	+		+		+	0.480	0.68	11	1206.49	0.09
-18.74	0.031	0.75	+	-0.0003	0.004	+		+			0.479	0.67	10	1206.77	0.08
-18.77	0.031	0.75	+	-0.0002		+		+	-0.0004		0.479	0.67	10	1206.91	0.07
**(Intercept)**	**Age**	**BMI**	**Traditional DP**	**Energy intake**	**Fiber**	**Gender**	**Physical activity**	**Place of residence**	**SFA**	**Smoking**	**R^2**	**adjR^2**	**df**	**AIC**	**Weight**
-18.91	0.029	0.74	+			+		+			0.478	0.67	8	1207.62	0.26
-19.21	0.031	0.75	+			+		+		+	0.479	0.67	10	1207.74	0.24
-18.79	0.029	0.74	+		-0.004	+		+			0.478	0.67	9	1209.27	0.11
-19.04	0.030	0.74	+			+		+	0.009		0.478	0.67	9	1209.42	0.10
-19.11	0.031	0.75	+		-0.003	+		+		+	0.479	0.67	11	1209.53	0.10
-19.32	0.031	0.75	+			+		+	0.008	+	0.479	0.67	11	1209.60	0.09
-18.89	0.029	0.74	+	-4.9E-06		+		+			0.478	0.67	9	1209.62	0.09

CVD – cardiovascular disease; BMI – body mass index; DP – dietary pattern; SFA – saturated fatty acids; AIC - Akaike information criterion

Table S8. OR calculated using averaged models for CVD prevalence in relation to dietary patterns adjusted for other predictors

Model	Variables in model	OR (95% CI)	p
CVD total prevalence vs Healthy Dietary Pattern	(Intercept)		0.0000
	Age	**1.10 (1.09-1.12)**	**0.0000**
	Healthy DP (L)	1.33 (0.98-1.79)	0.0654
	Healthy DP (C)	0.93 (0.71-1.22)	0.6009
	Healthy DP (Q)	1.06 (0.81-1.38)	0.6916
	Energy intake	1.00 (1.00-1.00)	0.2859
	Fiber	1.01 (0.99-1.03)	0.2886
	Gender (Male)	1.00 (0.75-1.34)	0.9959
	Place of residence (urban)	**0.56 (0.41-0.75)**	**0.0001**
	Saturated fatty acids	0.97 (0.93-1.02)	0.2359
	Smoking (past)	**1.59 (1.18-2.14)**	**0.0024**
	Smoking (current)	1.30 (0.89-1.89)	0.1800
CVD total prevalence vs Unhealthy Dietary Pattern	(Intercept)		0.0000
	Age	**1.10 (1.08-1.12)**	**0.0000**
	Unhealthy DP (L)	0.75 (0.55-1.03)	0.0747
	Unhealthy DP (C)	1.11 (0.85-1.46)	0.4273
	Unhealthy DP (Q)	1.06 (0.81-1.38)	0.6720
	Energy intake	1.00 (1.00-1.00)	0.5911
	Fiber	1.01 (1.00-1.02)	0.0856
	Gender (Male)	0.96 (0.72-1.27)	0.7781
	Place of residence (urban)	**0.56 (0.41-0.75)**	**0.0001**
	Saturated fatty acids	0.99 (0.95-1.03)	0.6512
	Smoking (past)	**1.55 (1.15-2.09)**	**0.0040**
	Smoking (current)	1.29 (0.88-1.88)	0.1905
CVD total prevalence vs Traditional Dietary Pattern	(Intercept)		0.00000
	Age	**1.10 (1.08-1.12)**	**0.00000**
	Traditional DP (L)	1.17 (0.86-1.61)	0.32040
	Traditional DP (C)	1.11 (0.86-1.44)	0.41531
	Traditional DP (Q)	0.88 (0.67-1.16)	0.36456
	Energy intake	1.00 (1.00-1.00)	0.07741
	Fiber	1.02 (1.00-1.04)	0.08419
	Gender (Male)	0.97 (0.73-1.29)	0.82571
	Place of residence (urban)	**0.62 (0.46-0.84)**	**0.00208**
	Saturated fatty acids	0.98 (0.94-1.03)	0.40671
	Smoking (past)	**1.55 (1.15-2.09)**	**0.00422**
	Smoking (current)	1.27 (0.87-1.86)	0.21531

CVD –cardiovascular disease; DP – dietary pattern; OR (95% CI) – odds ratio (95% confidence interval); L, Q, C - the coefficients of linear, quadratic and cubic relationships, respectively

Table S9. OR calculated using averaged models for hypertension prevalence in relation to dietary patterns adjusted for other predictors

Model	Variables in model	OR (95% CI)	p
Hypertension prevalence vs Healthy Dietary Pattern	(Intercept)		0.0000
	Age	**1.06 (1.05-1.07)**	**0.0000**
	BMI <18,5 kg/m^2	1.14 (0.37-3.54)	0.8244
	BMI 25-29,9 kg/m^2	**1.77 (1.40-2.24)**	**0.0000**
	BMI ≥30 kg/m^2	**3.38 (2.59-4.41)**	**0.0000**
	Healthy DP (L)	0.79 (0.62-1.01)	0.0611
	Healthy DP (C)	0.84 (0.69-1.02)	0.0864
	Healthy DP (Q)	1.03 (0.84-1.26)	0.7852
	Energy intake	1.00 (1.00-1.00)	0.1209
	Fiber	1.01 (1.00-1.03)	0.1366
	Gender (Male)	**2.68 (2.13-3.37)**	**0.0000**
	Place of residence (urban)	**1.25 (1.00-1.56)**	**0.0483**
	Saturated fatty acids	0.99 (0.96-1.02)	0.3764
	Smoking (past)	1.03 (0.82-1.30)	0.7878
	Smoking (current)	0.80 (0.62-1.03)	0.0895
Hypertension prevalence vs Unhealthy Dietary Pattern	(Intercept)		0.00000
	Age	**1.06 (1.05-1.07)**	**0.00000**
	BMI <18,5 kg/m^2	1.10 (0.36-3.36)	0.86467
	BMI 25-29,9 kg/m^2	**1.74 (1.37-2.20)**	**0.00000**
	BMI ≥30 kg/m^2	**3.27 (2.51-4.25)**	**0.00000**
	Unhealthy DP (L)	**0.77 (0.62-0.95)**	**0.01550**
	Unhealthy DP (C)	0.96 (0.79-1.17)	0.70046
	Unhealthy DP (Q)	1.11 (0.91-1.36)	0.29305
	Energy intake	1.00 (1.00-1.00)	0.52201
	Fiber	1.00 (1.00-1.01)	0.35735
	Gender (Male)	**2.87 (2.31-3.57)**	**0.00000**
	Place of residence (urban)	1.12 (0.90-1.39)	0.32531
	Saturated fatty acids	0.99 (0.96-1.02)	0.44780
	Smoking (past)	1.03 (0.81-1.30)	0.82203
	Smoking (current)	0.81 (0.63-1.05)	0.11627
Hypertension prevalence vs Traditional Dietary Pattern	(Intercept)		0.00000
	Age	**1.06 (1.05-1.07)**	**0.00000**
	BMI <18,5 kg/m^2	1.07 (0.35-3.28)	0.90916
	BMI 25-29,9 kg/m^2	**1.76 (1.39-2.23)**	**0.00000**
	BMI ≥30 kg/m^2	**3.37 (2.58-4.39)**	**0.00000**
	Traditional DP (L)	0.96 (0.77-1.19)	0.71454
	Traditional DP (C)	1.00 (0.82-1.22)	0.99471
	Traditional DP (Q)	1.06 (0.87-1.30)	0.54251
	Energy intake	1.00 (1.00-1.00)	0.16760
	Fiber	1.01 (1.00-1.02)	0.17104
	Gender (Male)	**2.85 (2.29-3.56)**	**0.00000**
	Place of residence (urban)	1.17 (0.94-1.45)	0.15252
	Saturated fatty acids	0.98 (0.95-1.01)	0.19032
	Smoking (past)	1.03 (0.82-1.31)	0.77616
	Smoking (current)	0.81 (0.63-1.05)	0.10516

CVD –cardiovascular disease; DP – dietary pattern; OR (95% CI) – odds ratio (95% confidence interval); BMI – body mass index; L, Q, C - the coefficients of linear, quadratic and cubic relationships, respectively

Table S10. OR calculated using averaged models for diabetes prevalence in relation to dietary patterns adjusted for other predictors

Model	Variables in model	OR (95% CI)	p
Diabetes prevalence vs Healthy Dietary Pattern	(Intercept)		0.00000
	Age	**1.06 (1.04-1.08)**	**0.00000**
	Healthy DP (L)	0.72 (0.48-1.07)	0.10792
	Healthy DP (C)	1.00 (0.72-1.39)	0.98100
	Healthy DP (Q)	1.19 (0.86-1.66)	0.29413
	Energy intake	**1.00 (1.00-1.00)**	**0.00121**
	Fiber	**1.05 (1.02-1.07)**	**0.00015**
	Gender (Male)	1.35 (0.94-1.92)	0.10317
	Place of residence (urban)	**0.21 (0.14-0.30)**	**0.00000**
	Saturated fatty acids	1.03 (0.98-1.09)	0.25210
	Smoking (past)	**1.59 (1.10-2.30)**	**0.01469**
	Smoking (current)	1.17 (0.74-1.83)	0.50527
Diabetes prevalence vs Unhealthy Dietary Pattern	(Intercept)		0.00000
	Age	**1.06 (1.04-1.08)**	**0.00000**
	Unhealthy DP (L)	**0.47 (0.31-0.70)**	**0.00026**
	Unhealthy DP (C)	0.80 (0.57-1.11)	0.17884
	Unhealthy DP (Q)	1.34 (0.96-1.85)	0.08238
	Energy intake	1.00 (1.00-1.00)	0.43835
	Fiber	1.02 (1.00-1.04)	0.06088
	Gender (Male)	1.33 (0.94-1.87)	0.10470
	Place of residence (urban)	**0.17 (0.12-0.25)**	**0.00000**
	Saturated fatty acids	1.01 (0.96-1.06)	0.71542
	Smoking (past)	**1.63 (1.12-2.37)**	**0.01113**
	Smoking (current)	1.21 (0.77-1.91)	0.40443
Diabetes prevalence vs Traditional Dietary Pattern	(Intercept)		0.00000
	Age	**1.06 (1.04-1.08)**	**0.00000**
	Traditional DP (L)	**2.00 (1.33-3.02)**	**0.00093**
	Traditional DP (C)	1.05 (0.77-1.44)	0.74857
	Traditional DP (Q)	1.00 (0.71-1.41)	0.99898
	Energy intake	**1.00 (1.00-1.00)**	**0.00006**
	Fiber	**1.04 (1.01-1.06)**	**0.00238**
	Gender (Male)	1.34 (0.95-1.90)	0.09423
	Place of residence (urban)	**0.21 (0.15-0.30)**	**0.00000**
	Saturated fatty acids	1.02 (0.97-1.08)	0.42720
	Smoking (past)	**1.59 (1.09-2.31)**	**0.01576**
	Smoking (current)	1.18 (0.75-1.86)	0.48068

CVD –cardiovascular disease; DP – dietary pattern; OR (95% CI) – odds ratio (95% confidence interval); L, Q, C - the coefficients of linear, quadratic and cubic relationships, respectively

Table S11. OR calculated using averaged models for impaired fasting glucose prevalence in relation to dietary patterns adjusted for other predictors

Model	Variables in model	OR (95% CI)	p
IFG prevalence vs Healthy Dietary Pattern	(Intercept)		0.00000
	Age	**1.02 (1.01-1.03)**	**0.00025**
	Healthy DP (L)	0.96 (0.76-1.22)	0.74915
	Healthy DP (C)	1.01 (0.81-1.26)	0.94157
	Healthy DP (Q)	1.09 (0.87-1.36)	0.46593
	Energy intake_kod	1.14 (0.90-1.45)	0.27547
	Fiber	1.00 (0.99-1.01)	0.52317
	Gender (Male)	1.00 (0.78-1.28)	0.99111
	Place of residence (urban)	**0.35 (0.28-0.44)**	**0.00000**
	Saturated fatty acids	0.99 (0.96-1.02)	0.53786
	Smoking (past)	**1.32 (1.02-1.70)**	**0.03695**
	Smoking (current)	**1.39 (1.04-1.85)**	**0.02733**
IFG prevalence vs Unhealthy Dietary Pattern	(Intercept)		0.00000
	Age	**1.02 (1.01-1.03)**	**0.00030**
	Unhealthy DP (L)	0.97 (0.75-1.26)	0.82719
	Unhealthy DP (C)	0.86 (0.69-1.08)	0.18673
	Unhealthy DP (Q)	1.19 (0.95-1.49)	0.12158
	Energy intake_kod	1.16 (0.89-1.51)	0.28539
	Fiber	1.00 (0.99-1.01)	0.65907
	Gender (Male)	1.00 (0.79-1.27)	0.98160
	Place of residence (urban)	**0.35 (0.27-0.44)**	**0.00000**
	Saturated fatty acids	0.99 (0.96-1.02)	0.50848
	Smoking (past)	**1.32 (1.02-1.71)**	**0.03407**
	Smoking (current)	**1.39 (1.04-1.85)**	**0.02549**
IFG prevalence vs Traditional Dietary Pattern	(Intercept)		0.00000
	Age	**1.02 (1.01-1.03)**	**0.00020**
	Traditional DP (L)	1.14 (0.90-1.44)	0.28332
	Traditional DP (C)	0.99 (0.79-1.24)	0.91353
	Traditional DP (Q)	**1.26 (1.00-1.58)**	**0.04730**
	Energy intake_kod	1.07 (0.83-1.37)	0.61988
	Fiber	1.00 (0.99-1.01)	0.74315
	Gender (Male)	1.00 (0.79-1.27)	0.99220
	Place of residence (urban)	**0.34 (0.27-0.44)**	**0.00000**
	Saturated fatty acids	0.99 (0.96-1.02)	0.54453
	Smoking (past)	**1.30 (1.01-1.69)**	**0.04546**
	Smoking (current)	**1.37 (1.03-1.83)**	**0.03265**

CVD –cardiovascular disease; DP – dietary pattern; OR (95% CI) – odds ratio (95% confidence interval); IFG – impaired fasting glucose; L, Q, C - the coefficients of linear, quadratic and cubic relationships, respectively; Energy intake_kod – categorical variable by median value

Table S12. OR calculated using averaged models for visceral obesity prevalence in relation to dietary patterns adjusted for other predictors

Model	Variables in model	OR (95% CI)	p
Visceral obesity prevalence vs Healthy Dietary Pattern	(Intercept)		0,00000
	Age	**1.03 (1.02-1.05)**	**0.00005**
	BMI	**2.12 (1.97-2.28)**	**0.00000**
	Healthy DP (L)	**0.68 (0.49-0.94)**	**0.02100**
	Healthy DP (C)	1.11 (0.83-1.48)	0.48976
	Healthy DP (Q)	0.83 (0.62-1.10)	0.19463
	Energy intake	1.00 (1.00-1.00)	0.42801
	Fiber	1.00 (0.99-1.02)	0.53434
	Gender (Male)	**0.24 (0.17-0.34)**	**0.00000**
	Place of residence (urban)	**0.32 (0.23-0.45)**	**0.00000**
	Saturated fatty acids	1.01 (0.96-1.05)	0.79784
	Smoking (past)	1.10 (0.78-1.54)	0.58726
	Smoking (current)	1.43 (0.98-2.08)	0.06657
Visceral obesity prevalence vs Unhealthy Dietary Pattern	(Intercept)		0.00000
	Age	**1.03 (1.02-1.05)**	**0.00003**
	BMI	**2.12 (1.97-2.28)**	**0.00000**
	Unhealthy DP (L)	1.44 (0.97-2.12)	0.06949
	Unhealthy DP (C)	1.08 (0.82-1.44)	0.57688
	Unhealthy DP (Q)	1.02 (0.76-1.37)	0.88244
	Energy intake	1.00 (1.00-1.00)	0.17693
	Fiber	1.00 (0.98-1.01)	0.70521
	Gender (Male)	**0.26 (0.19-0.37)**	**0.00000**
	Place of residence (urban)	**0.32 (0.23-0.45)**	**0.00000**
	Saturated fatty acids	1.00 (0.96-1.04)	0.98469
	Smoking (past)	1.12 (0.80-1.57)	0.52002
	Smoking (current)	1.45 (0.99-2.11)	0.05665
Visceral obesity prevalence vs Traditional Dietary Pattern	(Intercept)		0.00000
	Age	**1.03 (1.02-1.05)**	**0.00007**
	BMI	**2.11 (1.96-2.27)**	**0.00000**
	Traditional DP (L)	0.90 (0.66-1.23)	0.52298
	Traditional DP (C)	1.04 (0.78-1.40)	0.77353
	Traditional DP (Q)	1.07 (0.80-1.43)	0.63778
	Energy intake	1.00 (1.00-1.00)	0.96667
	Fiber	1.00 (0.98-1.01)	0.59990
	Gender (Male)	**0.28 (0.20-0.38)**	**0.00000**
	Place of residence (urban)	**0.29 (0.21-0.40)**	**0.00000**
	Saturated fatty acids	1.01 (0.97-1.05)	0.68189
	Smoking (past)	1.11 (0.79-1.55)	0.56101
	Smoking (current)	1.45 (1.00-2.12)	0.05177

CVD –cardiovascular disease; DP – dietary pattern; OR (95% CI) – odds ratio (95% confidence interval); BMI – body mass index; L, Q, C - the coefficients of linear, quadratic and cubic relationships, respectively

Table S13. OR calculated using averaged models for overweight and obesity prevalence in relation to dietary patterns adjusted for other predictors

Model	Variables in model	OR (95% CI)	p
Overweight + obesity prevalence vs Healthy Dietary Pattern	(Intercept)		0.0650
	Age_kod	**2.31 (1.88-2.84)**	**0.0000**
	Healthy DP (L)	1.06 (0.83-1.36)	0.6361
	Healthy DP (C)	0.96 (0.78-1.17)	0.6581
	Healthy DP (Q)	0.91 (0.74-1.12)	0.3766
	Energy intake	1.00 (1.00-1.00)	0.0677
	Fiber	1.01 (1.00-1.03)	0.0992
	Gender (Male)	**1.94 (1.53-2.46)**	**0.0000**
	Place of residence (urban)	**0.63 (0.50-0.80)**	**0.0001**
	Saturated fatty acids	1.01 (0.97-1.04)	0.7176
	Smoking (past)	**1.58 (1.24-2.01)**	**0.0002**
	Smoking (current)	0.89 (0.69-1.15)	0.3819
Overweight + obesity prevalence vs Unhealthy Dietary Pattern	(Intercept)		0.16776
	Age_kod	**2.28 (1.85-2.80)**	**0.00000**
	Unhealthy DP (L)	**0.72 (0.56-0.93)**	**0.01114**
	Unhealthy DP (C)	1.20 (0.98-1.46)	0.07436
	Unhealthy DP (Q)	**1.26 (1.03-1.54)**	**0.02613**
	Energy intake	1.00 (1.00-1.00)	0.74442
	Fiber	1.01 (1.00-1.02)	0.11156
	Gender (Male)	**1.89 (1.51-2.36)**	**0.00000**
	Place of residence (urban)	**0.61 (0.49-0.76)**	**0.00001**
	Saturated fatty acids	1.01 (0.98-1.04)	0.65290
	Smoking (past)	**1.55 (1.22-1.98)**	**0.00041**
	Smoking (current)	0.89 (0.69-1.14)	0.35180
Overweight + obesity prevalence vs Traditional Dietary Pattern	(Intercept)		0.002060
	Age_kod	**2.32 (1.89-2.86)**	**0.000000**
	Traditional DP (L)	**1.59 (1.24-2.03)**	**0.000220**
	Traditional DP (C)	0.88 (0.72-1.08)	0.209430
	Traditional DP (Q)	0.96 (0.78-1.18)	0.728380
	Energy intake	1.00 (1.00-1.00)	0.054550
	Fiber	1.01 (1.00-1.03)	0.093840
	Gender (Male)	**1.81 (1.44-2.27)**	**0.000000**
	Place of residence (urban)	**0.68 (0.54-0.86)**	**0.001140**
	Saturated fatty acids	1.01 (0.97-1.04)	0.691680
	Smoking (past)	**1.56 (1.22-1.99)**	**0.000370**
	Smoking (current)	0.88 (0.68-1.14)	0.345690

CVD –cardiovascular disease; DP – dietary pattern; OR (95% CI) – odds ratio (95% confidence interval); L, Q, C - the coefficients of linear, quadratic and cubic relationships, respectively; Age_kod – categorical variable by median value
